# Naturally Acquired Antibody Responses to *Plasmodium vivax* and *Plasmodium falciparum* Merozoite Surface Protein 1 (MSP1) C-Terminal 19 kDa Domains in an Area of Unstable Malaria Transmission in Southeast Asia

**DOI:** 10.1371/journal.pone.0151900

**Published:** 2016-03-21

**Authors:** Qinghui Wang, Zhenjun Zhao, Xuexing Zhang, Xuelian Li, Min Zhu, Peipei Li, Zhaoqing Yang, Ying Wang, Guiyun Yan, Hong Shang, Yaming Cao, Qi Fan, Liwang Cui

**Affiliations:** 1 Department of Immunology, College of Basic Medical Sciences, China Medical University, Shenyang, Liaoning, China; 2 Dalian Institute of Biotechnology, Dalian, Liaoning, China; 3 Department of Epidemiology, School of Public Health, China Medical University, Shenyang, Liaoning, China; 4 School of Humanities and Social Science, China Medical University, Shenyang, Liaoning, China; 5 Department of Pathogen Biology and Immunology, Kunming Medical University, Kunming, China; 6 Institute of Tropical Medicine, Third Military Medical University, Chongqing, China; 7 Program in Public Health, University of California Irvine, Irvine, CA, United States of America; 8 Department of Laboratory Medicine, the First Hospital of China Medical University, Shenyang, Liaoning, China; 9 Department of Entomology, Pennsylvania State University, 501 ASI Building, University Park, PA, 16802, United States of America; Liverpool School of Tropical Medicine, UNITED KINGDOM

## Abstract

Understanding naturally acquired immunity to infections caused by *Plasmodia* in different malaria endemicity settings is needed for better vaccine designs and for exploring antibody responses as a proxy marker of malaria transmission intensity. This study investigated the sero-epidemiology of malaria along the international border between China and Myanmar, where malaria elimination action plans are in place. This study recruited 233 *P*. *vivax* and 156 *P*. *falciparum* infected subjects with acute malaria at the malaria clinics and hospitals. In addition, 93 and 67 healthy individuals from the same endemic region or from non-endemic region, respectively, were used as controls. Acute malaria infections were identified by microscopy. Anti-recombinant PfMSP1_19_ and PvMSP1_19_ antibody levels were measured by ELISA. Antibody responses to respective MSP1_19_ were detected in 50.9% and 78.2% patients with acute *P*. *vivax* and *P*. *falciparum* infections, respectively. There were cross-reacting antibodies in *Plasmodium* patients against these two recombinant proteins, though we could not exclude the possibility of submicroscopic mixed-species infections. IgG1, IgG3 and IgG4 were the major subclasses. Interestingly, 43.2% of the healthy endemic population also had antibodies against PfMSP1_19_, whereas only 3.9% of this population had antibodies against PvMSP1_19_. Higher antibody levels were correlated with age and parasite density, but not with season, gender or malaria history. Both total IgG and individual IgG subclasses underwent substantial declines during the convalescent period in three months. This study demonstrated that individuals in a hypoendemic area with coexistence of *P*. *vivax* and *P*. *falciparum* can mount rapid antibody responses against both PfMSP1_19_ and PvMSP1_19_. The significantly higher proportion of responders to PfMSP1_19_ in the healthy endemic population indicates higher prevalence of *P*. *falciparum* in the recent past. Specific antibodies against PvMSP1_19_ could serve as a marker of recent exposure to *P*. *vivax* in epidemiological studies.

## Introduction

Malaria still remains one major infectious disease worldwide, despite that intensive efforts have been undertaken to overcome this ancient foe. According to the 2014 World Malaria Report [1], an estimated 198 million malaria cases and 584,000 deaths occurred in 2013. Malaria vaccines are considered an important strategy to prevent and eliminate *Plasmodium* infections. However, numerous challenges including genetic diversity of *Plasmodium* vaccine candidates and short persistence of anti-parasite immunity hinder vaccine development.

Antibody responses against malaria parasite antigens have been extensively studied [2]. Naturally acquired antibodies against individual antigens or panels of antigens in hyperendemic regions have been associated with protection against clinical disease and severity [3–6]. However, the associations between antibodies against parasite antigens and risk of malaria are not always consistent [2], which may depend on parasite antigens [7] and vary considerably between different malaria-endemic areas. Since epidemiological and environmental factors such as *Plasmodium* species, host genetics and behaviors all affect the development of immunity against malaria parasites, detailed profiling of naturally acquired antibodies directed against parasite antigens in different malaria endemic regions will provide useful information for vaccine design. In many endemic areas, more than one *Plasmodium* parasite species infects humans. Interactions occur between different parasite species [8], and as a result, prior infections by one species influence the course of a subsequent infection by the same or a different species [9]. Antigens with high levels of homology between malaria parasite species may elicit cross-reactive antibodies targeting more than one parasite species [10–12]. Thus, antibody responses to individual antigens may evolve differently, depending on the epidemiological settings. In addition, it is commonly believed that acquired antibodies to malaria is short lived and require periodic reinfections to maintain [13]. Thus, the prevalence and intensity of antibody responses may be used as proxy measures of transmission intensity [14]. Serological markers are predicted to be particularly useful in areas of unstable malaria transmission.

Merozoite surface protein1 (MSP1), a highly conserved protein among *Plasmodium* species as well as the most abundant protein expressed on the surface of merozoites, is a leading vaccine candidate[15,16]. MSP1 is synthesized as a ~200 kDa precursor protein attached to the merozoite surface via a C-terminal anchor, and later processed into four major fragments prior to schizont rupture. Subsequently, one processed product, the MSP1_42_ C-terminal fragment, experiences further cleavage into MSP1_33_ and MSP1_19_ portions during merozoite invasion into an erythrocyte. Finally, MSP1_33_ is released into circulation and MSP1_19_ is the only fragment that remains on merozoite surface, which is detectable in the newly invaded erythrocyte [17–19]. The MSP1_19_ fragment is localized in the highly conserved C-terminus. Several studies have demonstrated that MSP1_19_ is highly immunogenic in both animal and human infections [20–23]. Naturally acquired antibodies against MSP1_19_ can inhibit parasite growth *in vitro* [18,24] and are associated with the protective immunity against malaria infection [25–28].

In the Greater Mekong Subregion (GMS) of Southeast Asia, malaria exhibits enormous geographical heterogeneity and complexity with the coexistence of *P*. *vivax* and *P*. *falciparum* [29]. In recent years, extensive control efforts have led to a significant reduction in parasite prevalence and changing malaria epidemiology. One noticeable change is the increasing proportion of *P*. *vivax* malaria, a species that is more difficult to eliminate. As several nations in this area are pursuing malaria elimination, a better understanding of the changing malaria epidemiology will enable the design and deployment of more effective control measures. Here we tried to determine the prevalence of antibody responses against the MSP1_19_ antigens of *P*. *falciparum* (PfMSP1_19_) and *P*. *vivax* (PvMSP1_19_) to explore their potentials as serological markers for epidemiological studies in a low-endemicity area along the China-Myanmar border. In addition, we measured the levels of naturally induced IgG subclasses to these antigens as an indication of the functionality of the antibody responses.

## Materials and Methods

### Study area, subjects and blood sample collection

This study was conducted at the China-Myanmar border area (97.56° E and 24.75° N), where malaria burden remains high among the ethnic minorities (mostly Kachin or Jingpo) residing in this region [29]. Malaria transmission here is perennial but seasonal with most of the malaria cases occurring in the rainy season from May through October [30]. *P*. *vivax* and *P*. *falciparum* coexist here and *P*. *vivax* has become more prevalent. This study aimed to investigate the prevalence of antibody responses against recombinant MSP1_19_ proteins in malaria patients. In 2011–2013, we enrolled a total of 389 patients with acute malaria infections through passive case detection of malaria patients attending two local clinics and a township hospital (47, 157 and 172 patients, respectively), and active case detection in five local villages and two settlements for internally displaced people (totally 13 additional patients). Written informed consent was obtained from all participants/legal guardians before enrolment, and assents were also obtained from patients 7–14 years. Enrolled patients were interviewed by trained medical personnel, who used questionnaire to obtain demographic and epidemiological information. Malaria infections were diagnosed by microscopic examination of both thin and thick blood films. Patients showing signs of severe malnutrition, pregnancy (verbally affirmed), and underlying diseases were excluded. Only patients infected with a single *Plasmodium* species were included in the analysis. Peripheral blood samples (2–3 ml) from participants were obtained by venipuncture into EDTA tubes before administration of treatment, kept on ice and transferred to the nearby field laboratory on the same day for processing. Blood samples were obtained from 27 patients up to 3 months in order to follow the dynamics of antibody titers. For comparison, 2 ml of blood samples were also obtained from 93 healthy individuals living in the same endemic region and 67 healthy individuals from a non-endemic area (Shenyang, China). The study received ethical approval from the Institutional Review Board of Pennsylvania State University, USA, Institutional Review Board of Kunming Medical University, China, and Bioethics Committee of the Bureau of Health of Kachin, Myanmar.

### Laboratory procedures

Plasma and blood cells were separated by centrifugation and stored separately at -80°C. Thin and thick blood smears were read by two experienced microscopists to confirm parasite species. Parasite density was estimated by counting the number of asexual parasites and gametocytes per 200 leukocytes assuming 8,000 WBCs/μL of blood.

### Expression and purification of recombinant MSP1_19_ proteins

Both MSP1_19_ proteins were expressed using established methods, which allow the expression of correctly folded recombinant proteins [27,31,32]. The PfMSP1_19_ fragment corresponding to amino acids 1609–1702 was amplified using genomic DNA of *P*. *falciparum* 3D7 with forward primer 5’-CTGGATCCATTTCACAACACCAATGCGT-3’ (*Bam*HI site underlined) and reverse primer 5’-GTCTCGAGGTTAGAGGAACTGCAGAAAATAC-3’ (*Xho*I site underlined). The PvMSP1_19_ fragment corresponding to amino acids 1636–1746 of PVX_099980 of the Sal I strain was amplified from the genomic DNA of a *P*. *vivax* field isolate using forward primer 5’-CTGGATCCACTCAGTTATTAACTATGAGCT-3’ (*Bam*HI site underlined) and reverse primer 5’-GTCTCGAGGAGGAAAAGCAACATGAGCAAC-3’ (*Xho*I site underlined). The PfMSP1_19_ was cloned into the *Bam*HI-*Xho*I sites of the expression vector pET32a (Novagen) to obtain the pET32a/PfMSP1_19_ construct. The PvMSP1_19_ was cloned into the *Bam*HI-*Xho*I sites of pGEX-6P-1(GE Healthcare) in frame with the glutathione S-transferase (GST) tag at its N terminus to obtain the pGEX-6P-1/PvMSP1_19_ construct. Both constructs were transformed into *Escherichia coli* BL21 (DE3) strain (Novagen) for protein expression. For PfMSP1_19_, protein expression was induced with 1 mM isopropyl-β-D-thiogalactopyranoside (IPTG) for 3 h at 37°C. The recombinant PfMSP1_19_ was purified under denaturing conditions using Ni-NTA His·Bind Resins (Novagen). The purified recombinant PfMSP1_19_ was refolded by dialyzing in phosphate buffered saline (PBS, pH 7.4) with a urea gradient (from 4 to 0 M). Finally, the purified recombinant PfMSP1_19_ was dialyzed against 10% glycerol (v/v) in PBS (pH7.4). For PvMSP1_19_, the protein expression was induced with 0.1 mM IPTG for 4 h at 37°C. The recombinant PvMSP1_19_ was purified under native conditions using Glutathione Sepharose 4B (GE Healthcare) column previously equilibrated with PBS (pH 7.4). The tagged protein bound to the column was washed with 10 bed volumes of PreScission cleavage buffer (50 mM Tris-HCl, 150 mM NaCl, 1 mM EDTA, pH 7.5), and digested in gel with PreScission™ (GE Healthcare) at 4°C for 4 h. Following incubation, the column was washed with 3 bed volumes of PreScission cleavage buffer, and the eluate was collected in different tubes and analyzed by SDS-PAGE. The eluates containing PvMSP1_19_ were pooled and dialyzed against 10% glycerol (v/v) in PBS (pH 7.4). Following purification, the protein concentrations were determined by the Bradford assay (Bio-Rad). Recombinant proteins were separated on 15% SDS-PAGE under both reducing and non-reducing conditions to determine whether proteins had the correct folding.

### Enzyme-linked immunosorbent assay (ELISA)

The plasma samples were analyzed by ELISA for the detection of naturally acquired antibodies against recombinant MSP1_19_. In brief, 96-well flat-bottom microplates (Corning, NY) were pre-coated with 0.5 μg recombinant MSP-1_19_ (either PfMSP-1_19_ or PvMSP-1_19_) per well and incubated overnight at 4°C. After blocking with PBS containing 1% BSA for 2 h, 100 μL of diluted samples (1:200 for total IgG, and 1:50 for IgG subclasses) per well were added and incubated at room temperature for 2 h. The plates were washed with PBST (PBS containing 0.05% Tween 20) for five times, and incubated with the peroxidase-conjugated goat anti-human IgG or IgG subsets (Sigma, St. Louis, MO) for 2 h. Subsequently, the wash step was repeated, and the plate was developed with substrate reagent pack (R&D Systems, Minneapolis, MN) for 15 min. The reaction was stopped by adding sulfuric acid and the optical density (OD) at 450 nm was determined using a plate reader. The cutoff value was defined as the average of nonendemic control (NC) samples plus two standard deviations (0.635, 0.225, 0.964, 0.225, and 0.175 for IgG, IgG1, IgG2, IgG3, and IgG4, respectively for PvMSP1_19_; 0.182, 0.144, 0.217, 0.137, and 0.167 for IgG, IgG1, IgG2, IgG3, and IgG4, respectively for PfMSP1_19_). Positive samples confirmed by preliminary experiment and negative samples from non-endemic region were included in each plate as controls. The OD ratio was referred to the observed OD value of tested sample divided by the value of the cutoff as used in other studies (e.g., [33,34]). OD ratio ≥ 1.0 was considered positive.

### Sequence analysis

*P*. *falciparum or P*. *vivax* DNA was extracted from filter papers or whole blood collected from the patients using QIAamp DNA Blood Mini kit (QIAGEN, Hilden, Germany) according to the manufacturer’s instructions. The regions encoding *PfMSP1*_*19*_ and *PvMSP1*_*19*_ were amplified with the following primer pairs: *PfMSP1*_*19*_ forward (TCACAACACCAATGCGTAAAA) and reverse (GAGTATTAATAAGAATGATATTCCTAAG); and *PvMSP1*_*19*_ forward (ACCAATGTGGCTGATAATGC) and reverse (TCAAAGAGTGGCTCAGAACC). Each 20 μl of PCR mixture contained 11.7 μl sterile water, 2 μl of 10×KOD-Plus-Neo buffer, 0.8 μl MgSO_4_ (25 mM), 2 μl dNTP mixture (2 mM), 1.0 μl of each primers (10 μM), 0.5 μl KOD PLUS-Neo DNA polymerase (1Unit/μl) (Toyobo, Japan) and 1 μl template DNA. Cycling conditions were as follows: 94°C for 2 min, 40 cycles of 94°C for 15 sec, 56°C for 15 sec, and 68°C for 1 min, and then a final extension at 68°C for 5 min. The PCR products were purified using the QIAquick Gel Extraction Kit (QIAGEN, CA, USA) and sequenced with the PCR primers in both directions (BGI Tech Solutions Co., Ltd.). MSP1 fragments from 45 *P*. *falciparum* and 76 *P*. *vivax* monoclonal infections were successfully sequenced. To evaluate the polymorphism of *PMSP1*_*19*_ gene, the MSP1 gene of *P*. *falciparum* 3D7 strain *or P*. *vivax* Sal-I strain was used as the references. The sequences were aligned using the CLUSTALW program in MEGA 6.0.

### Statistical analysis

Data were analyzed by the software of SPSS 13.0 or GraphPad Prism 5. Normality was tested by Kolmogorov-Smirnov test. If the data did not follow a normal distribution, the data were analyzed using nonparametric methods. Differences in the level of IgG and IgG subclasses among more than two groups were analyzed by one-way Kruskal-Wallis test and Dunn's test, whilst differences between two groups were compared by Mann-Whitney *U* test. The χ^2^ test was used to compare the percentages of demographic and clinical data between patients with acute *P*. *falciparum* and *P*. *vivax* infections as well as the prevalence of PMSP1_19_ IgG positivity among different groups. Logistic regression was applied to compare the prevalence of positive antibody responses. Spearman’s rank correlation test was performed to analyze the correlations between total IgG and its subclasses (log-transformed) and the correlation between antibody levels and tracing time. Dynamic changes of antibodies were estimated via linear regression of OD ratio against the time of sampling. *p*<0.05 was considered significant.

## Results

### Demographic and clinical features of acute malaria infections

From June 2011 through December 2013, 389 patients in northeast Myanmar with acute malaria were recruited to participate in this study (Table 1). Since these clinics and hospital are within a 5 km of radius and serve overlapping catchment areas, we pooled these samples for analysis so that the data were representative of the local malaria epidemiology. Among these malaria patients, 233 and 156 were microscopy-positive for *P*. *vivax* or *P*. *falciparum* infections, respectively. The majority of the participants (93.8%) were ethnic Kachin. The enrolled subjects had a median age of 19 (ranged 1–82 years), and 65.3% were male. Twelve (3.1%) patients had a previous malaria infection within the past 12 months. At enrolment, 78.7% of the patients were febrile (axillary temperature >37.5°C), and 75.3% of the patients sought treatment within three days of fever history. Overall, patients had a median parasite density of 3,400 asexual parasites/μl with only two patients having parasite densities exceeding 100,000/μl, and 35.5% of the patients presented with gametocytemia.

**Table 1:** Demographic and clinical features of patients with acute *P*. *falciparum* and *P*. *vivax* infections.

	Total	*P*. *falciparum*	*P*. *vivax*
Number of patients	389	156	233
Kachin ethnicity [N (%)]	365 (93.8%)	146 (93.6%)	219 (94.0%)
Gender			
Male [N (%)]	254(65.3%)	111(71.2%)	143(61.4%)*
Female [N (%)]	135(34.7%)	45(28.8%)	90(38.6%)*
Age (years) (median/range)	19 (1–82)	23 (1–71)	13 (1–82)***
Under 5 years [N (%)]	37 (9.5%)	3 (1.9%)	34 (14.6%)***
5–14 years [N (%)]	108 (27.8%)	19 (12.2%)	89 (38.2%)***
≥15 years [N (%)]	244 (62.7%)	134 (85.9%)	110 (47.2%)***
Number of patients by season: [N (%)]			
Rainy (May-October)	320 (82.3%)	149 (95.5%)	171 (73.4%)***
Dry (November-April)	69 (17.7%)	7 (4.5%)	62 (26.6%)***
Patients with malaria infection during the past 12 months [N (%)]	12 (3.1%)	8 (5.1%)	4 (1.7%)
Febrile patients on day 0 (axillary temperature >37.5°C) [N (%)]	306 (78.7%)	119 (76.3%)	187 (80.3%)
Axillary temperature (°C) [mean (range)]	38.5 (36.0–43.0)	38.4 (36.0–43.0)	38.5 (36.0–40.6)
Days with fever before seeking treatment: [N (%)]			
1	28 (7.2%)	11(7.1%)	17(7.3%)
2	113 (29.0%)	50(32.1%)	63(27.0%)
3	152 (39.1%)	44(28.2%)	108(46.4%)***
4	42 (10.8%)	21(13.5%)	21(9.0%)
>4	35(9.0%)	17(10.9%)	18(7.7%)
Unknown	19 (4.9%)	13(8.3%)	6(2.6%)**
Asexual parasite density [median (IQR)]	3,400 (840–10,160)	2680(800–13,180)	3440(1,100–9,260)
Patients with gametocytes on day 0 [N (%)]	138 (35.5%)	9 (5.7%)	129 (55.4%)***
# Gametocyte density [geometric mean (range)]	238 (40–1,680)	121 (40–1,280)	250 (40–1,680)***

^#^ Four (2 *P*. *falciparum* and 2 *P*. *vivax*) patients only had gametocytes (80–1280 gametocytes/μl).

* Indicates significant difference between *P*. *falciparum* and *P*. *vivax* cases at *P* = 0.05 (Mann-Whitney U test or χ^2^ test).

** Indicates significant difference between *P*. *falciparum* and *P*. *vivax* cases at *P* = 0.01 (Mann-Whitney U test or χ^2^ test).

*** Indicates significant difference between *P*. *falciparum* and *P*. *vivax* cases at *P* = 0.001 (Mann-Whitney U test or χ^2^ test).

When comparing the demographic data between *P*. *vivax* and *P*. *falciparum* cases, we identified several significant differences (Table 1). Whereas both groups were male-biased, the median age of *P*. *falciparum* patients (23 years) were significantly higher than that of the *P*. *vivax* patients (13 years) (*p*<0.01, Mann-Whitney *U* test), consistent with earlier findings of a much higher risk of children of 5–14 years for having *P*. *vivax* infections and a higher risk of adults for having *P*. *falciparum* infections [30]. Although both parasite species displayed apparent seasonality, their seasonal dynamics were radically different (χ^2^ = 31.34, *p*<0.001). The overwhelming majority of *P*. *falciparum* cases (>95%) were detected in the rainy season, whereas >25% of *P*. *vivax* infections also occurred in the dry season. For both *P*. *falciparum* and *P*. *vivax* cases, most patients sought treatment with less than three days of fever history, but significantly more *P*. *vivax* patients had three days of fever history. Totally 12 cases reported previous infections with *Plasmodium* during the past 12 months. Interestingly, whereas asexual parasitemias of *P*. *falciparum* and *P*. *vivax* patients did not differ significantly at enrolment, a significantly higher proportion of *vivax* patients presented with gametocytes (χ^2^ = 102, *p*<0.001). Moreover, gametocyte density was significantly higher in *P*. *vivax* patients (*p*<0.01, Mann-Whitney *U* test).

### Naturally acquired antibody responses against MSP1_19_

**Fig 1:**
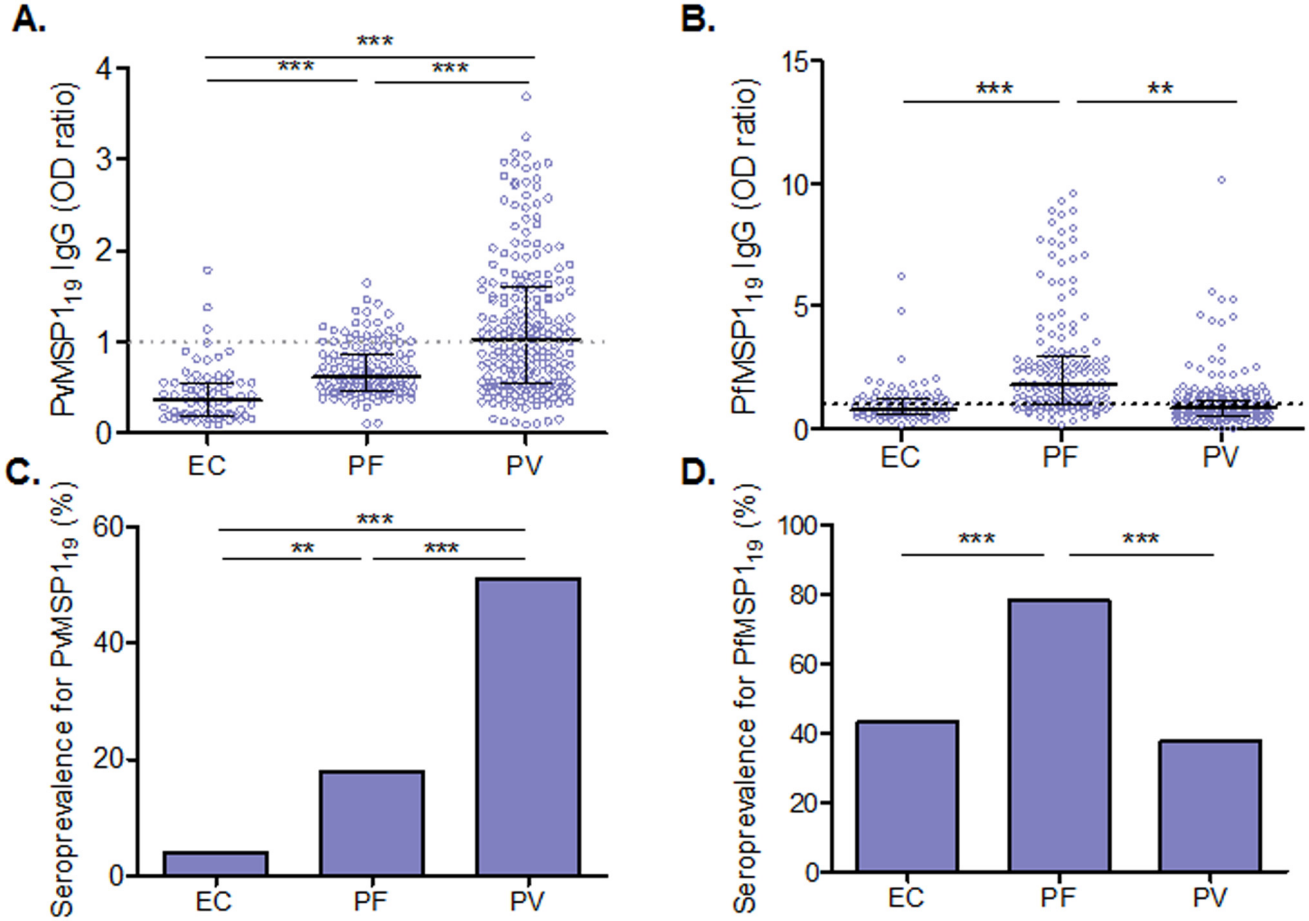
Natural antibody responses to recombinant PvMSP1_19_ (A and C) and PfMSP1_19_ (B and D) antigens. Plasma samples from healthy endemic control (EC), *P*. *falciparum* patients (PF) and *P*. *vivax* patients (PV) were used in PvMSP1_19_ or PfMSP1_19_ ELISA, respectively. **A** and **B**: IgG levels in these samples for PvMSP1_19_ (**A)** and PfMSP1_19_ **(B)**. Data shown as median ± interquartile range were analyzed by one-way nonparametric Kruskal-Wallis test and Dunn's test for multiple comparisons. **C** and **D**: Prevalence of IgG positive samples for PvMSP1_19_ **(C)** and PfMSP1_19_ **(D)**. Data were analyzed by χ^2^ test. OD cutoff value was defined as the average of non-endemic control samples plus two standard deviations. OD ratio was referred to the observed OD value of a test sample divided by the cutoff value. OD ratio ≥ 1.0 was considered positive (above the threshold shown as dashed line at 1). ** and *** indicate significance at *p*<0.01 and *p*<0.001, respectively.

We investigated the prevalence of naturally acquired antibodies against *Plasmodium* MSP1_19_ in this *P*. *falciparum* and *P*. *vivax* coexisting, low-endemicity area. Both recombinant PfMSP1_19_ and PvMSP1_19_ were expressed in *E*. *coli* and purified to almost homogeneity as shown in SDS-PAGE gels (S1 Fig). The proteins migrated differentially under denaturing (+DTT) and non-reducing (-DTT) conditions, suggesting the formation of disulfide bonds in the recombinant proteins. These recombinant proteins were used in ELISA to determine the presence of naturally acquired antibodies in malaria patients. Compared to healthy local inhabitants as the endemic control (EC) group, both *P*. *falciparum* and *P*. *vivax* patients contained significantly higher antibody levels against PvMSP1_19_ and PfMSP1_19_ (*p* < 0.001, one-way non-parametric Kruskal-Wallis test or Dunn's test for multiple comparisons), indicating significant induction of antibodies against MSP1 during acute malaria infections (Fig 1A and 1B). In addition, ~19% of patients with acute infections had positive antibodies against both PfMSP1_19_ and PvMSp1_19_, suggesting the presence of cross-reacting antibodies. However, the levels and frequencies of antibody responses to MSP1_19_ differed substantially between *P*. *falciparum* and *P*. *vivax* patients (Fig 1). As expected, *P*. *vivax* patients had significantly higher antibody levels to PvMSP1_19_ than *P*. *falciparum* patients, and vice versa (Fig 1A and 1B). Compared to the baseline antibody levels in healthy individuals from a non-endemic area, the prevalence of responders to PvMSP1_19_ was 3.9%, 18.0% and 50.9% in the EC group, *P*. *falciparum* and *P*. *vivax* patients, respectively (Fig 1C). Likewise, the prevalence of responders to PfMSP1_19_ was 43.2%, 78.2% and 37.7% in the EC group, *P*. *falciparum* and *P*. *vivax* patients, respectively (Fig 1D). Logistic regression analysis showed that acute *P*. *vivax* patients were 25.19 times more likely to have PvMSP1_19_-specific IgG than the EC group (Table 2), whereas *P*. *falciparum* patients were 4.72 times more likely to have PfMSP1_19_-specific IgG than the EC group (Table 2). Yet, it is noteworthy that proportion of PfMSP1_19_ responders in the EC group (43.2%) was significantly higher than that of PvMSP1_19_ responders (3.9%) (χ^2^ = 33.48, *p*<0.0001).

**Table 2:** Logistic regression predicting positive vs negative IgG responses anti-MSP1_19_.

Groups	IgG against PfMSP1_19_			IgG against PvMSP1_19_		
	OR	95% CI	*p*-value	OR	95% CI	*p*-value
*P*. *vivax*	0.8	0.48∼1.32	0.38	25.19	7.72∼82.21	<0.0001****
*P*. *falciparum*	4.72	2.67∼8.33	<0.0001****	5.34	1.56∼18.31	0.008**

OR: Odds ratio.

** Indicates significance at *p* <0.01.

**** Indicates significance at p <0.0001.

### MSP1_19_–specific IgG subclasses

**Fig 2:**
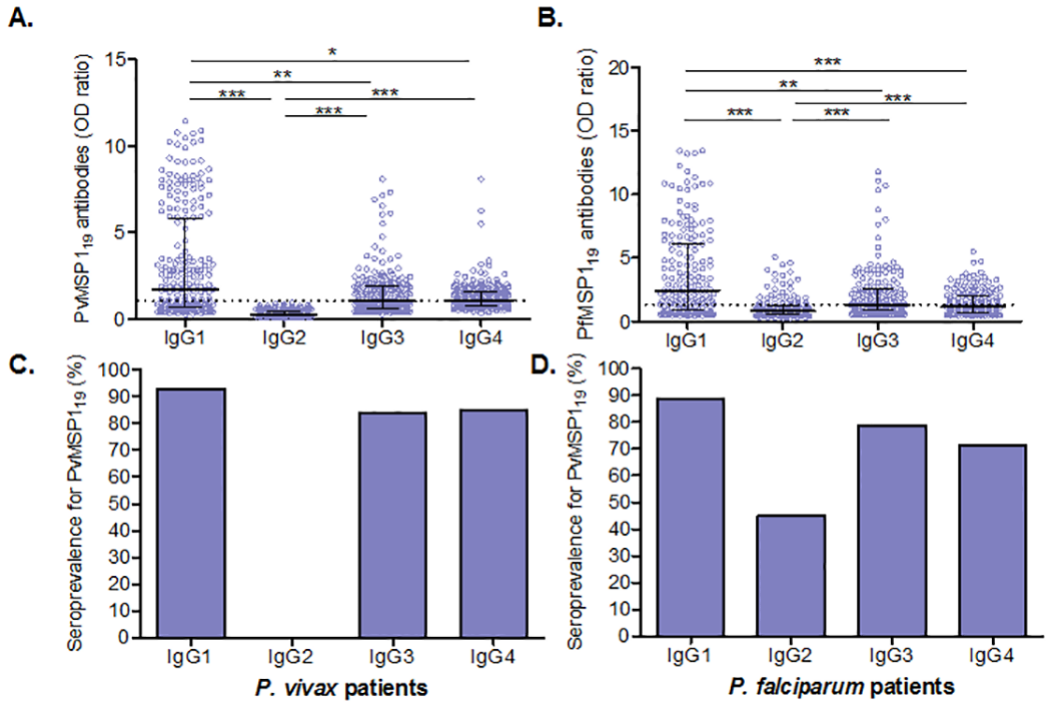
IgG subclass responses to acute *P*. *vivax* (A and C) *or P*. *falciparum* (B and D) infections. **A** and **B**: Levels of IgG subclasses in samples from acute *P*. *vivax* or *P*. *falciparum* patients against respective PvMSP1_19_ **(A)** and PfMSP1_19_ **(B)**. Data shown as median ± interquartile range were analyzed by one-way nonparametric Kruskal-Wallis test and Dunn's test for multiple comparisons. C and D: Prevalence of IgG subclasses against PvMSP1_19_ in IgG-positive *P*. *vivax* patients **(C)** and against PfMSP1_19_ in IgG-positive *P*. *falciparum* patients **(D)**. OD cutoff value and OD ratio were defined as in Fig 1. OD ratio ≥ 1.0 was considered positive (above the threshold shown as dashed line at 1). *, ** and *** indicate significance at *p*<0.05, *p*<0.01 and *p*<0.001, respectively.

**Fig 3:**
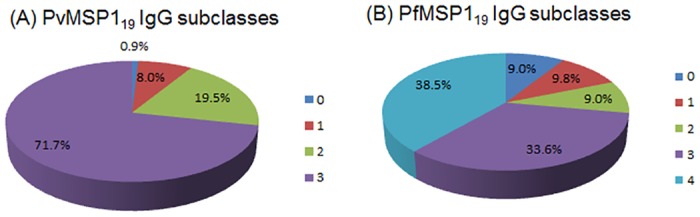
Cumulative positivity of patients’ plasma samples for IgG subclasses against PvMSP1_19_ (A) and PfMSP1_19_ (B). IgG-positive samples were stratified by their positivity for any of the IgG subclasses. Data are plotted as the percentages of *P*. *vivax* or *P*. *falciparum* patients postivie for 0–4 IgG subclasses to PvMSP1_19_ **(A)** and PfMSP1_19_ **(B)**. The five portions (0, 1, 2, 3, and 4) denote the seropositvitiy for 0, 1, 2, 3, and 4 IgG subclasses to the respective MSP1_19_.

We have subsequently profiled antibody responses in malaria patients against MSP1_19_ by the four IgG subclasses (Fig 2). Compared to the EC group, patients with acute *Plasmodium* infections had much higher levels of IgG subclasses. For both *P*. *vivax* and *P*. *falciparum* patient groups against their respective MSP1_19_, the levels of IgG subclasses differed significantly (Fig 2A and 2B) (*p* < 0.05, one-way Kruskal-Wallis test and Dunn’s test). IgG1 levels were the highest, followed by IgG3 and IgG4. Among samples positive for total IgG (IgG responders), *P*. *falciparum* patients showed the lowest IgG2 responses to PfMSP1_19_, whereas positive IgG2 responses to PvMSP1_19_ were not detected in *P*. *vivax* patients (Fig 2). These results showed that IgG1 and IgG3 subclasses were the predominant antibody responses during *P*. *vivax* and *P*. *falciparum* infections. When IgG responders were stratified by the positivity to any of the IgG subclasses, 0.9% of *P*. *vivax* patients lacked antibody responses to PvMSP1_19_, whereas 71.7% simultaneously had IgG1, IgG3 and IgG4 to PvMSP1_19_ (Fig 3A). Similarly, 9.0% of *P*. *falciparum* patients had no antibodies to PfMSP1_19_ in any of the IgG subclasses, while 72.1% of *P*. *falciparum* patients had IgGs in three or more IgG isotypes to PfMSP1_19_ (Fig 3B). Spearman’s rank correlation test detected a significant positive correlation between the magnitudes of total IgG level and each IgG subclass, with the highest correlation found for IgG1 (r = 0.78 for PvMSP1_19_ and r = 0.74 for PfMSP1_19_) and IgG3 (r = 0.71 for both PvMSP1_19_ and PfMSP1_19_) (S2 Fig).

### Factors associated with variations in antibody responses

**Fig 4:**
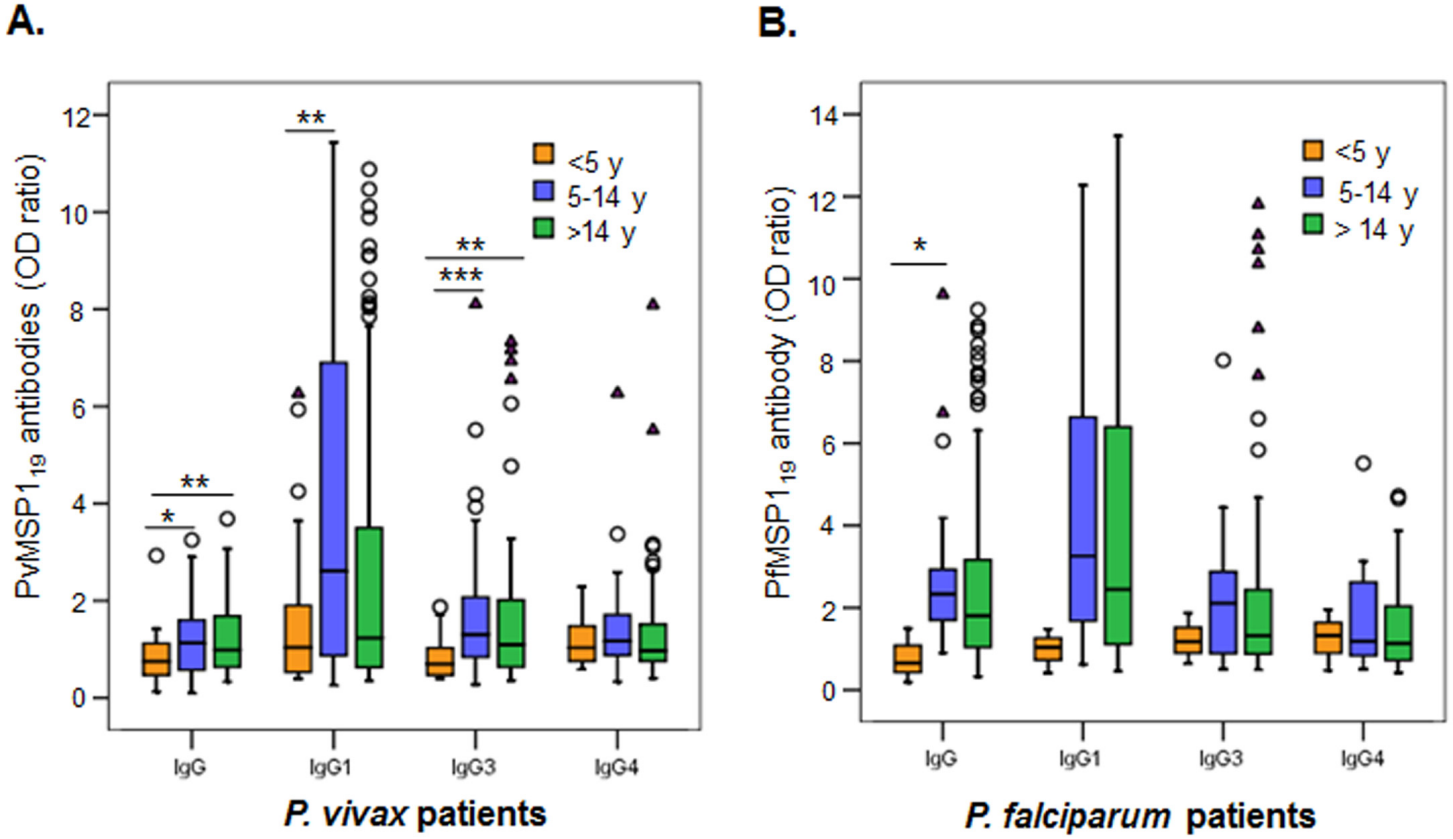
Antibody responses in acute *P*. *vivax* (A) and *P*. *falciparum* (B) patients of different ages. Patients were stratified into under 5 years old (<5 y), 5–14 years old (5–14 y) and more than 14 years old (>14 y) groups. Data are shown in box plots with median as a line within the box and interquartile value at the edge of box. The range of the column was 1.5 times of interquartile range. Any outlier values exceeding 1.5 and 3 times of the interquartile range were shown as circles and triangles, respectively. Data were analyzed by one-way nonparametric Kruskal-Wallis test and Dunn's test for multiple comparisons. *, ** and *** indicate significance at *p*<0.05, *p*<0.01 and *p*<0.001, respectively.

**Fig 5:**
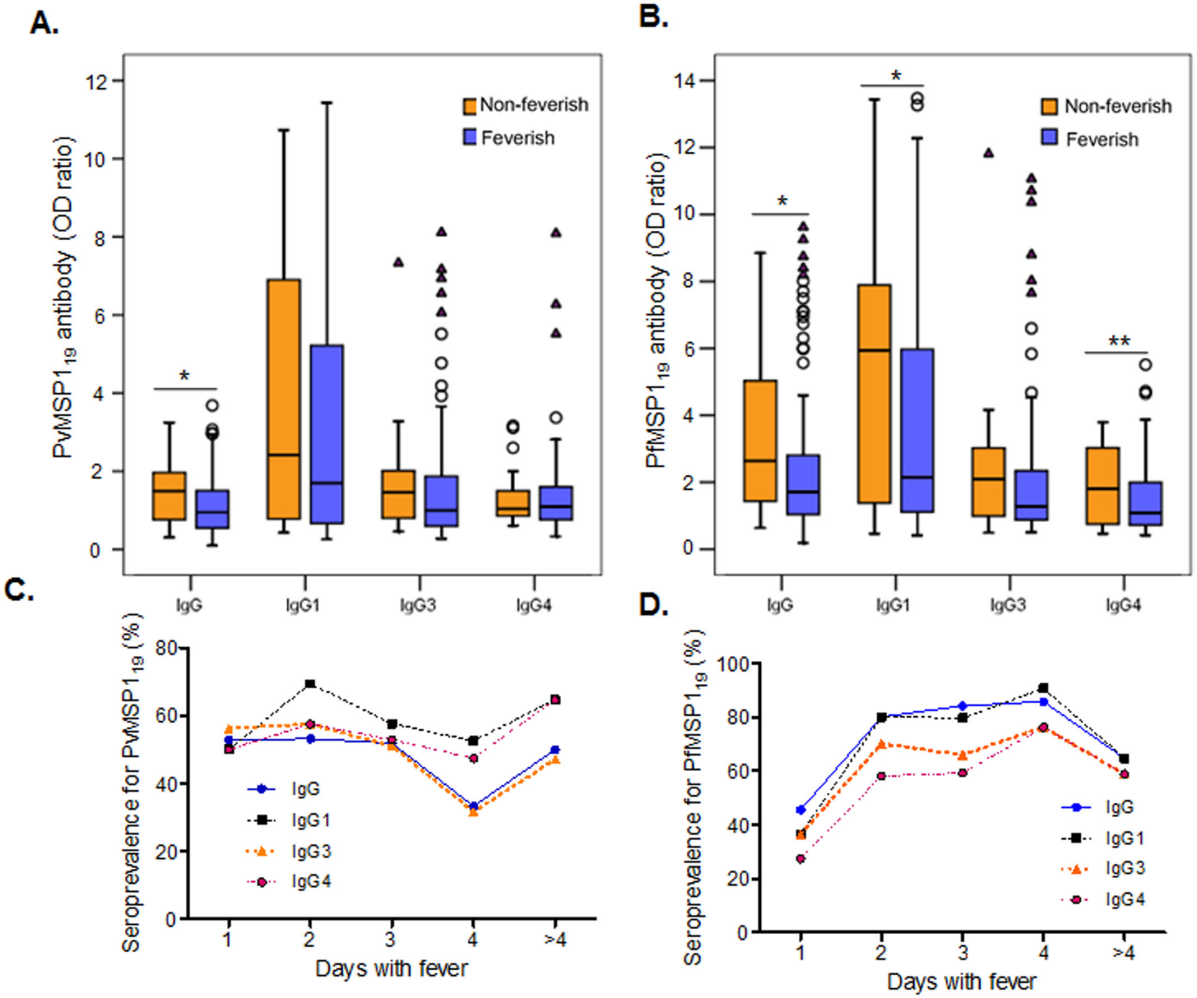
Antibody responses in acute *P*. *vivax* (A) and *P*. *falciparum* (B) patients presented with or without fever and with different fever histories. **A** and **B:** Patients were stratified into non-febrile (axillary temperature <37.5°C) and febrile (≥37.5°C) groups and antibody levels against PvMSP1_19_ (**A**) and PfMSP1_19_ (**B**) were compared. Data are presented in box plots with the median shown as a line within the box and interquartile value at the edge of box. The whole range of the column was 1.5 times of interquartile range. Any outlier values exceeding 1.5 and 3 times of the interquartile range are shown as circles and triangles, respectively. Data were analyzed by Mann-Whitney’s *U* test. * and ** indicate significance at *p*<0.05 and *p*<0.01, respectively. **C** and **D:** Seroprevalence against PvMSP1_19_ (**C**) and PfMSP1_19_ (**D**) in patients with different fever histories. Patients were stratified by the recorded numer of days patients experienced fever before seeking treatment (Days with fever) (1–4 and more than 4 days).

**Fig 6:**
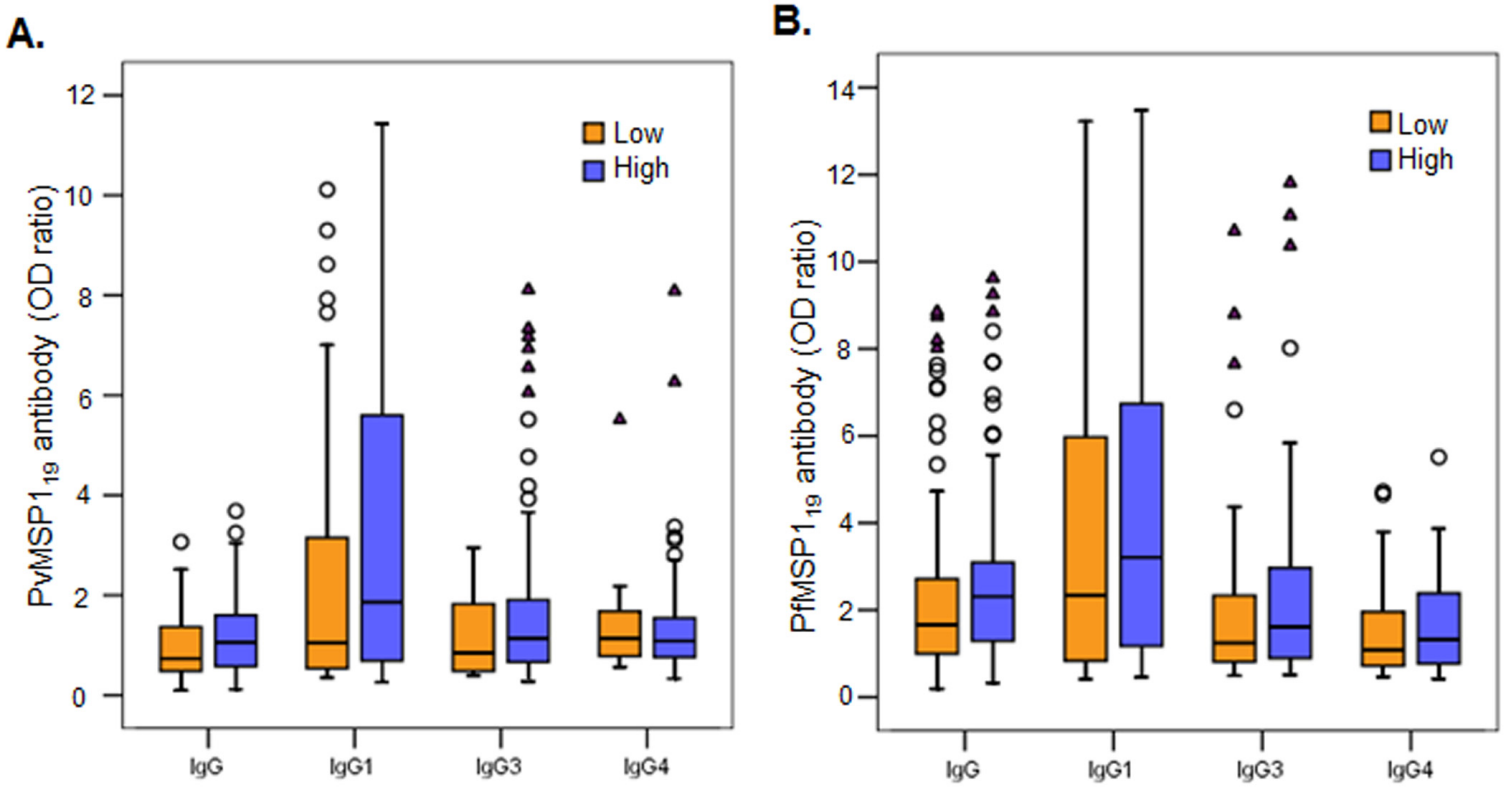
Antibody responses in acute *P*. *vivax* (A) and *P*. *falciparum* (B) patients with different level of asexual parasitemias. Patients were stratified into low (<500 parasites/μl for *P*. *vivax* or <5000 parasites/μl for *P*. *falciparum*) and high (≥ 500 parasites/μl for *P*. *vivax* or ≥ 5000 parasites/μl for *P*. *falciparum*) parasitemia groups. Data are presented in box plots with the median shown as a line within the box and interquartile value at the edge of box. The range of the column was 1.5 times of interquartile range. Any outlier values exceeding 1.5 and 3 times of the interquartile range are shown as circles and triangles, respectively. Data were analyzed by Mann-Whitney’s *U* test.

We then analyzed potential factors contributing to the variations in MSP1_19_ antibodies during acute *Plasmodium* infections. Based on the information collected from the surveys, patients were stratified by age, gender, parasitemia, previous infection history, and season. Analysis was restricted to total IgG and three subclasses (IgG1, IgG3 and IgG4). Despite that malaria in the endemic settings displayed clear seasonality and >80% of samples were collected during the rainy season (May-October), seroprevalence did not differ between the dry and rainy seasons (S3 Fig). Among the age groups, both *P*. *vivax* and *P*. *falciparum* patients younger than five years had the least levels of IgG to MSP1_19_ and the lowest seroprevalence (Fig 4, Table 3). In both groups, IgG1 and IgG3 subclasses appeared to have contributed the most to the age-dependent difference (Fig 4, Table 3). It is interesting to note that there was a trend towards higher antibody levels in the 5–14 years group than in the >14 years group, albeit the differences were not statistically significant (Fig 4). Gender and previous infection history did not show evident impact on the antibody responses (S4 and S5 Figs). All patients with fever at the time of enrolment had lower total IgG levels to respective MSP1_19_ as well as individual IgG subclasses (IgG1, 3, and 4) than those without fever (Fig 5). Febrile *P*. *vivax* patients had significantly lower total IgG to PvMSP1_19_ than non-febrile patients, whereas febrile *P*. *falciparum* patients had significantly lower IgG, IgG1, and IgG4 levels than those without fever (Fig 5). For fever history (the number of days patients experienced fever before seeking treatment), seroprevalence in *P*. *falciparum* patients gradually increased as the days with fever increased during the first four days, whereas seroprevalence in *P*. *vivax* patients did not show such a trend (Fig 5). When patients were stratified based on the presence of low (<500 parasites/μl for *P*. *vivax* or <5000 parasites/μl for *P*. *falciparum*) and high asexual parasite densities (≥500 parasites/μl for *P*. *vivax* or ≥5000 parasites/μl for *P*. *falciparum*), higher IgG, IgG1, and IgG3 levels were associated with the high density group, albeit the difference between the low and high parasite density groups was not significant (Fig 6). Similarly, the proportions of responders to the respective MSP1_19_ were also higher in the high parasite density groups (Table 4).

**Table 3:** Proportions of responders (%) to recombinant MSP1_19_ from different age groups.

	IgG			IgG1			IgG3			IgG4		
	<5 y	5–14 y	≥15 y	<5 y	5–14 y	≥15 y	<5 y	5–14 y	≥15 y	<5 y	5–14 y	≥15 y
PvMSP1_19_	38.2	57.3	49.5	52.9	70.8	54.1	26.5	60.7	51.0	50.0	65.2	48.0
PfMSP1_19_	33.3	89.5	77.6	66.7	84.2	75.4	66.7	68.4	65.7	66.7	63.2	58.2

**Table 4:** Proportions of responders (%) to recombinant MSP1_19_ in the patient groups with low and high parasite densities.

	IgG		IgG1		IgG3		IgG4	
	low	high	low	high	low	high	low	high
PvMSP1_19_	37.1	53.3	51.6	62.1	38.7	53.2	54.8	55.3
PfMSP1_19_	74.2	83.6	73.0	80.6	65.2	67.2	53.9	65.7

### Dynamics of antibody levels during convalescence

**Fig 7:**
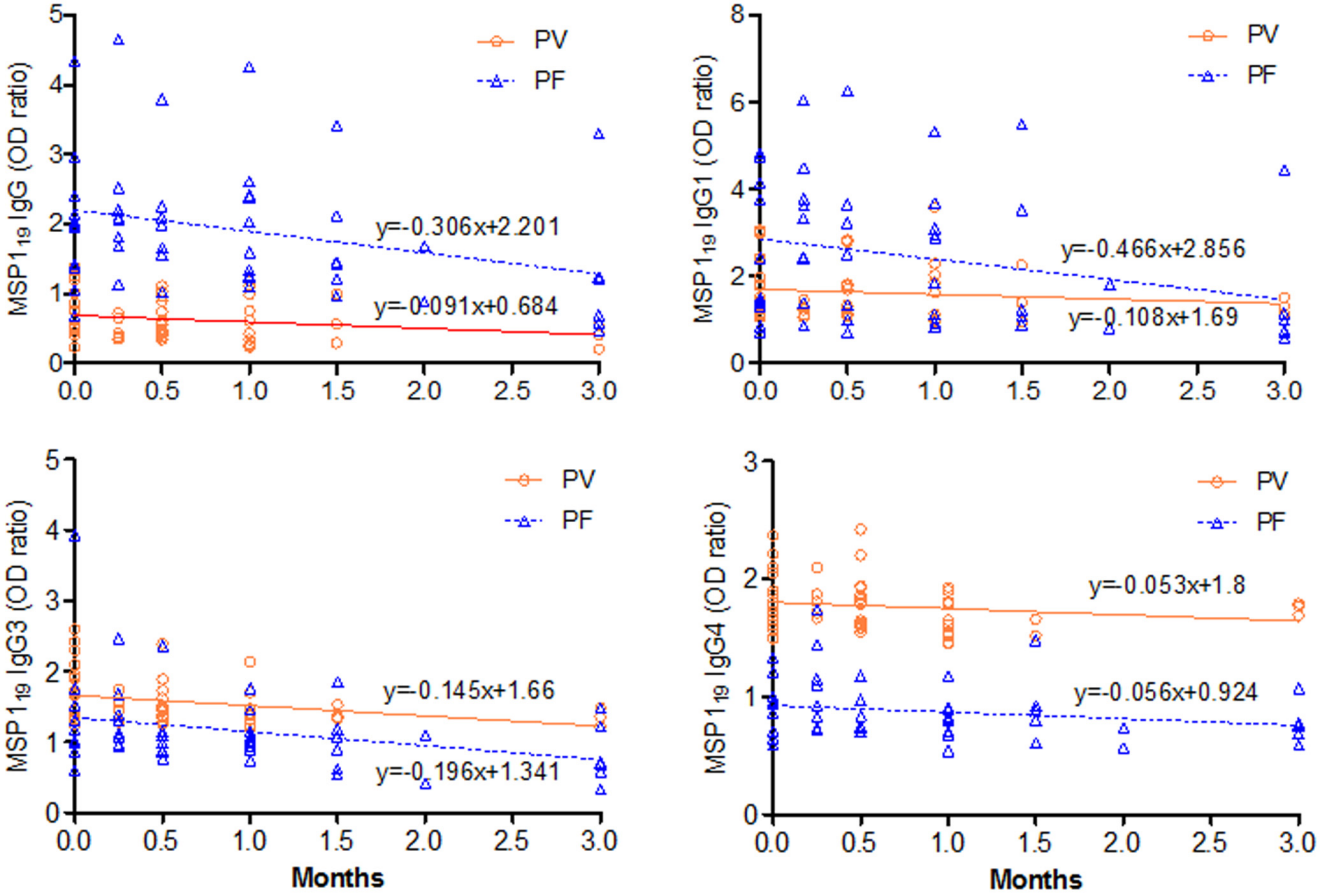
Dynamic decays of MSP1_19_ antibodies within 3 months of follow up. *P*. *vivax* (PV) or *P*. *falciparum* (PF) infected patients were followed to determine the dynamics of antibody levels for three months since enrolment. The dynamic changes of antibody levels were estimated via linear regression.

With 27 patients enrolled for follow-ups, only five completed the three-month follow-ups. In both *P*. *vivax* and *P*. *falciparum* patients, total IgG, IgG1 and IgG3 levels all declined substantially during the follow-up period (Fig 7). Linear regression analysis showed significant decay rates of total IgG (*β* = 0.306, *p* = 0.030) and IgG3 (*β* = 0.196, *p* = 0.021) antibodies in *P*. *falciparum* patients and IgG3 (*β* = 0.145, *p* = 0.024) levels in *P*. *vivax* patients over the three months of follow-up. In all cases, IgG, IgG1 and IgG3 antibodies declined relatively faster in *P*. *falciparum* patients than in *P*. *vivax* patients.

### PfMSP1_19_ and PvMSP1_19_ sequence variations

We sequenced PfMSP1_19_ fragments from 45 available *P*. *falciparum* isolates in the study samples and found that the predominant haplotypes E-KNG and E-TSR (present in the 3D7 clone) were found in 29 and 11 samples (S6 Fig), respectively, which is similar to E-KNG, E-TSR and Q-KNG being the predominant haplotypes in adjacent Yunnan province of China [35]. Genotyping PvMSP1_19_ fragments from 76 *P*. *vivax* isolates in the study samples revealed only one allele type, which is the same as in the Sal-I strain.

## Discussion

Naturally acquired antibodies against *Plasmodium* merozoite surface antigens play a major role in protection against malaria. MSP1 is one of the most abundant and highly immunogenic merozoite surface antigens. Antibodies to both PfMSP1 and PvMSP1 are highly prevalent in malaria endemic populations [22,36–42]. Strong antibody responses to PfMSP1_19_ were associated with protection against clinical malaria and disease severity in hyperendemic areas [4,6,28,43], whereas antibodies against the PvMSP1 N-terminal variable region was associated with reduced risk of *P*. *vivax* infection and clinical protection [44,45]. Even in low-endemic areas with unstable malaria transmission, antibody responses to merozoite antigens are highly prevalent in individuals with acute malaria [38,46–49]. Here we investigated antibody responses to recombinant MSP1_19_ in acute malaria patients in a hypoendemic area of Southeast Asia where both *P*. *vivax* and *P*. *falciparum* are prevalent. Consistent with earlier findings [50,51], considerable induction of antibody responses to respective MSP1_19_ were detected in 50.9% *P*. *vivax* and 78.2% *P*. *falciparum* patients. This robust induction of antibody responses could be resulted from boosting of antibody production via activation of antigen-specific memory B cells from previous exposures [52]. Yet, one striking observation is that 43.2% of the healthy residents of this endemic area had IgG responses to PfMSP1_19_, whereas only 3.9% of them had antibodies to PvMSP1_19_. This is slightly different from the high prevalence of IgG responders to both PfMSP1_19_ (52%) and PvMSP1_19_ (70%) in a South American region with a similar endemicity setting of coexistence of *P*. *vivax* and *P*. *falciparum* malaria [53]. With the evidence that both antibodies and memory B cells to malaria antigens could be stably maintained over time in the absence of reinfection even in areas of extremely low transmission [54], the persistence of high levels of IgG responses detected in the healthy endemic population could be resulted from long-lasting antibodies [52]. Given that antibodies to both antigens exhibited relatively paralleled decay rates over time, the disparate responders to PvMSP1_19_ and PfMSP1_19_ in the healthy residents of the endemic area may correspond to the changing malaria epidemiology from *P*. *falciparum* to *P*. *vivax* dominance in recent years [30]. Under such a scenario, the significantly higher IgG responders in the endemic healthy participants to PfMSP1_19_ may indicate exposures to more intensive *P*. *falciparum* transmission in the recent past. In addition, it has been shown that PvMSP1_19_ is highly immunogenic and can elicit a rapid humoral response in acute *P*. *vivax* infections [38,46,49]. Thus, the high seroprevalence to PvMSP1_19_ among *P*. *vivax* patients in our study was most likely resulted from the current infections.

In areas of co-endemicity of multiple malaria parasite species, homologous antigens may elicit cross-reactive antibodies [10–12]. We detected that 18% of patients with acute *P*. *falciparum* infections had detectable antibodies against PvMSP1_19_, which might represent cross-reactive antibodies elicited by *P*. *falciparum* infections, although we could not exclude the possible presence of submicroscopic *P*. *vivax* infections. The MSP1_19_ fragments of the two species share ~50% amino acid identity and may possess common B cell epitopes. In addition, the presence of different variants of the PfMSP1_19_ fragment can lead to variant-specific antibody responses [55,56]. Though the PfMSP1_19_ polymorphism does not appear to restrict antibody recognition to the entire domain [57,58], the significance of variant-specific antibodies requires further investigation [59]. In comparison, the monoallelic PvMSP1_19_ indicates that sequence polymorphism in the PvMSP1_19_ fragment does not play a significant role in the varied antibody responses in different individuals in our endemic site.

The subclasses of IgG with different structures mediate different immune effector functions. The cytophilic subclasses IgG1 and IgG3, the predominant subclasses produced to merozoite antigens, play an important role in opsonization and complement-mediated lysis of the merozoites [60–63]. In contrast, the non-cytophilic IgG2 and IgG4 subclasses, which may compete with cytophilic antibodies for antigen recognition, are normally associated with susceptibility to *P*. *falciparum* malaria [27,64,65]. Our studies demonstrated significant induction of IgG1 and IgG3 antibodies to the MSP1_19_, a finding consistent with results from most malaria endemic areas [27,28,63,66,67], including other endemic areas in the GMS [50,51]. Since IgG1/IgG3 class switching may be affected by the nature of the antigen, exposures and host factors [63], there are numerous studies documenting differential prevalence of these two cytophilic classes [36,67–70]. In our case, both the proportions of responders and the magnitudes of IgG1 and IgG3 levels were comparable between *P*. *vivax* and *P*. *falciparum* patients to their respective MSP1_19_. While the magnitudes and proportions of responders of IgG2 responses were low or non-detectable in patients, more than half of the patients contained IgG4 antibodies. As detected in earlier studies in the GMS [50,51], such high percentages of IgG4 responders may imply general susceptibility of the people in this region to repeated *Plasmodium* infections [71]. Specifically, 71.7% IgG-positive *P*. *vivax* and 72.1% IgG-positive *P*. *falciparum* patients contained three and more IgG subclasses to their respective MSP1_19_ antigens. Although the reasons for this interesting IgG subclass pattern are not clear, it might be attributable to host genetic background, transmission intensity and other demographic and epidemiological factors. Of note, host cytokines such as IL-10 and IFN-γ could profoundly affect the malaria parasite-specific IgG3 and IgG4 [34,72–75].

In areas with seasonal malaria transmission, antibody levels often fluctuate with substantial increases in the high season when infections are prevalent and subsequent declines after the infections are resolved [52,76]. In some areas, such a seasonal fluctuation may not be very evident [40], probably as a result of maintenance of antibodies from past infections. In our analysis, we did not find a clear difference in seroprevalence between high and low transmission seasons. Whereas this could indicate persistence of antibodies from earlier infections, it could also be due to the design of this study, which measured antibody responses in individuals with acute malaria infections. In this case, robust induction and boosting of antibody responses might have occurred, which might have obscured the baseline antibody levels with possible seasonal difference. This possibility will be addressed in future studies targeting the entire endemic population. Furthermore, a much larger sample size from the dry season is needed for a more robust conclusion. In addition, significant boosting of antibody response normally occurs in patients with a recent malaria history (e.g., <6 months) [52,77]. The small number of patients with recent malaria history in our study precluded a robust correlation analysis.

It is widely accepted that development of protective immune responses requires repeated exposure to malaria, and as a result older people in endemic areas tend to have higher antibody levels. Increased prevalence of antibodies against merozoite surface proteins such as MSP1 with age has been documented in various endemic settings [3,28,36,37,76], and our results are highly agreeable with this earlier conclusion. Our study, however, showed that the 5–14 years age group even had higher IgG1 and IgG3 antibodies than the >14 years group, suggesting that this age group may have experienced boosting of the immune responses from more intense malaria exposure. This agrees well with the result of our recent epidemiological investigation in the same region, where we found that 5–14 year-old school children tended to have about twice the odds of having vivax malaria [30]. Since high antibody titers against blood stage antigens before infection are associated with clinical protection, antibody titers are often inversely correlated with parasite density [40,55,76]. In our analysis, we found higher, albeit insignificant, levels of total IgG and subclasses in patients with higher parasitemias during acute infections. It is likely that in this malaria hypoendemic area, the low baseline antibody titers might be too low to be protective against malaria infection or disease severity. Besides, antibodies against PfMSP1_19_ failed to show clinical protection in a hyperendemic area of Myanmar [50]. Higher parasite density may even induce higher antibody responses in patients with acute malaria infection. If true, antibodies against MSP1_19_ may serve as an indicator of recent *Plasmodium* infections.

In conclusion, this immuno-epidemiological study conducted in the malaria hypoendemic area along the China-Myanmar border reveals several interesting findings. Both acute *P*. *falciparum* and *P*. *vivax* infections had age-dependent elicitation of antibody responses in patients and the cytophilic IgG1 and IgG3 were the predominant subclasses. In addition, the induction of IgG4 suggests the overall antibody profile in these patients may not be protective against infections. In the healthy endemic population, IgG response to PfMSP1_19_ attained 43.2% prevalence, whereas seroprevalence to PvMSP1_19_ was only 3.9%. Though some extents of cross-reactivity may exist between PfMSP1_19_ and PvMSP1_19_, the transient induction of PvMSP1_19_ antibodies during acute *P*. *vivax* infection, substantial antibody decay during convalescence, and low baseline seroprevalence altogether suggest the antibodies to PvMSP1_19_ may serve as a serological marker for malaria transmission in the study area.

## Supporting Information

S1 Fig Expression and purification of recombinant PfMSP1_19_ and PvMSP1_19_. Recombinant proteins were separated on 15% SDS-PAGE under reducing (+DTT) and nonreducing (-DTT) conditions and stained with Coomassie blue.
(PDF)

S2 Fig Correlations between antibody responses of total IgG and its subclasses specific against PvMSP1_19_ (A) and PfMSP1_19_ (B) in acute patients. Data were log transformed and Spearman’s rank correlation tests were performed. All comparisons were significantly different with *p*<0.0001. r: Spearman's correlation coefficient.
(PDF)

S3 Fig Distribution and seroprevalence of *P*. *vivax* (A) and *P*. *falciparum* (B) patients in different months. Bars represent the number of cases in which orange and blue bars are IgG negative and IgG positive, respectively. Lines represent seroprevalence.
(PDF)

S4 Fig Antibody responses in acute *P*. *vivax* (A) and *P*. *falciparum* (B) infected patients of different genders. Data are presented in box plots with the median shown as a line within the box and interquartile value at the edge of box. The range of the column was 1.5 times of interquartile range. Any outlier values exceeding 1.5 and 3 times of the interquartile range are shown as circles and triangles, respectively. Data were analyzed by Mann-Whitney’s U test.
(PDF)

S5 Fig Antibody responses in acute *P*. *vivax* (A) and *P*. *falciparum* (B) infected patients with or without previous *Plasmodium* infection history. Data are presented in box plots with the median shown as a line within the box and interquartile value at the edge of box. The range of the column was 1.5 times of interquartile range. Any outlier values exceeding 1.5 and 3 times of the interquartile range are shown as circles and triangles, respectively. Data were analyzed by Mann-Whitney’s U test.
(PDF)

S6 Fig PfMSP1_19_ amino acid sequences from 45 available *P*. *falciparum* samples. **(A)** Alignment of the 5 haplotypes (H1 –H5) with the reference 3D7 sequence. Residue substitutions are shadowed in red. **(B)** Frequencies of the five haplotypes.
(PDF)
