# Supplementary figures and images for: Naturally Acquired Antibody Responses to Plasmodium vivax and Plasmodium falciparum Merozoite Surface Protein 1 (MSP1) C-Terminal 19 kDa Domains in an Area of Unstable Malaria Transmission in Southeast Asia
Source: PLoS One. 2016 Mar 21;11(3):e0151900. doi: 10.1371/journal.pone.0151900 (PMC4801383; doi:10.1371/journal.pone.0151900)

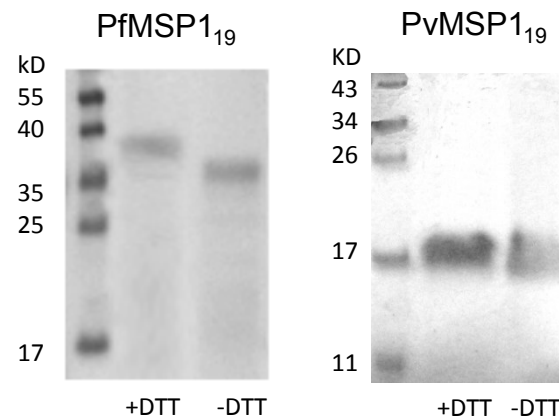

**S1 Fig. Expression and purification of recombinant PfMSP1<sub>19</sub> and PvMSP1<sub>19</sub>.**

Supplement: S1 Fig — Recombinant proteins were separated on 15% SDS-PAGE under reducing (+DTT) and nonreducing (-DTT) conditions and stained with Coomassie blue. (PDF) [file pone.0151900.s001.pdf]
