# Supplementary material for: Naturally Acquired Antibody Responses to Plasmodium vivax and Plasmodium falciparum Merozoite Surface Protein 1 (MSP1) C-Terminal 19 kDa Domains in an Area of Unstable Malaria Transmission in Southeast Asia
Source: PLoS One. 2016 Mar 21;11(3):e0151900. doi: 10.1371/journal.pone.0151900 (PMC4801383; doi:10.1371/journal.pone.0151900)
Supplement: S2 Fig — Data were log transformed and Spearman’s rank correlation tests were performed. All comparisons were significantly different with p<0.0001. r: Spearman's correlation coefficient. (PDF) [file pone.0151900.s002.pdf]

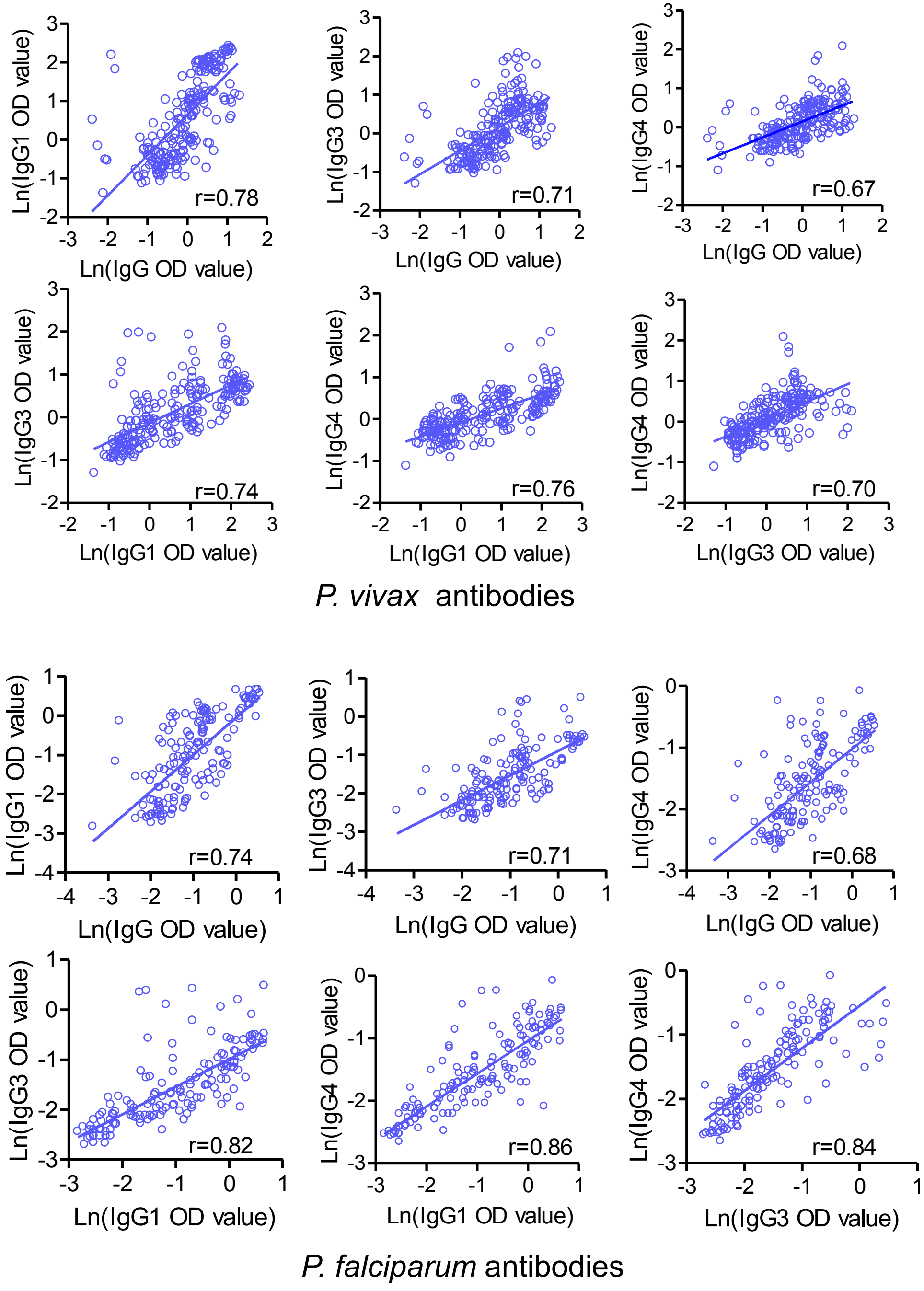

S2 Fig. Correlations between antibody responses of total IgG and its subclasses specific against PvMSP1$_{19}$ (A) and PfMSP1$_{19}$ (B) in acute patients.
