# Supplementary material for: Naturally Acquired Antibody Responses to Plasmodium vivax and Plasmodium falciparum Merozoite Surface Protein 1 (MSP1) C-Terminal 19 kDa Domains in an Area of Unstable Malaria Transmission in Southeast Asia
Source: PLoS One. 2016 Mar 21;11(3):e0151900. doi: 10.1371/journal.pone.0151900 (PMC4801383; doi:10.1371/journal.pone.0151900)
Supplement: S3 Fig — Bars represent the number of cases in which orange and blue bars are IgG negative and IgG positive, respectively. Lines represent seroprevalence. (PDF) [file pone.0151900.s003.pdf]

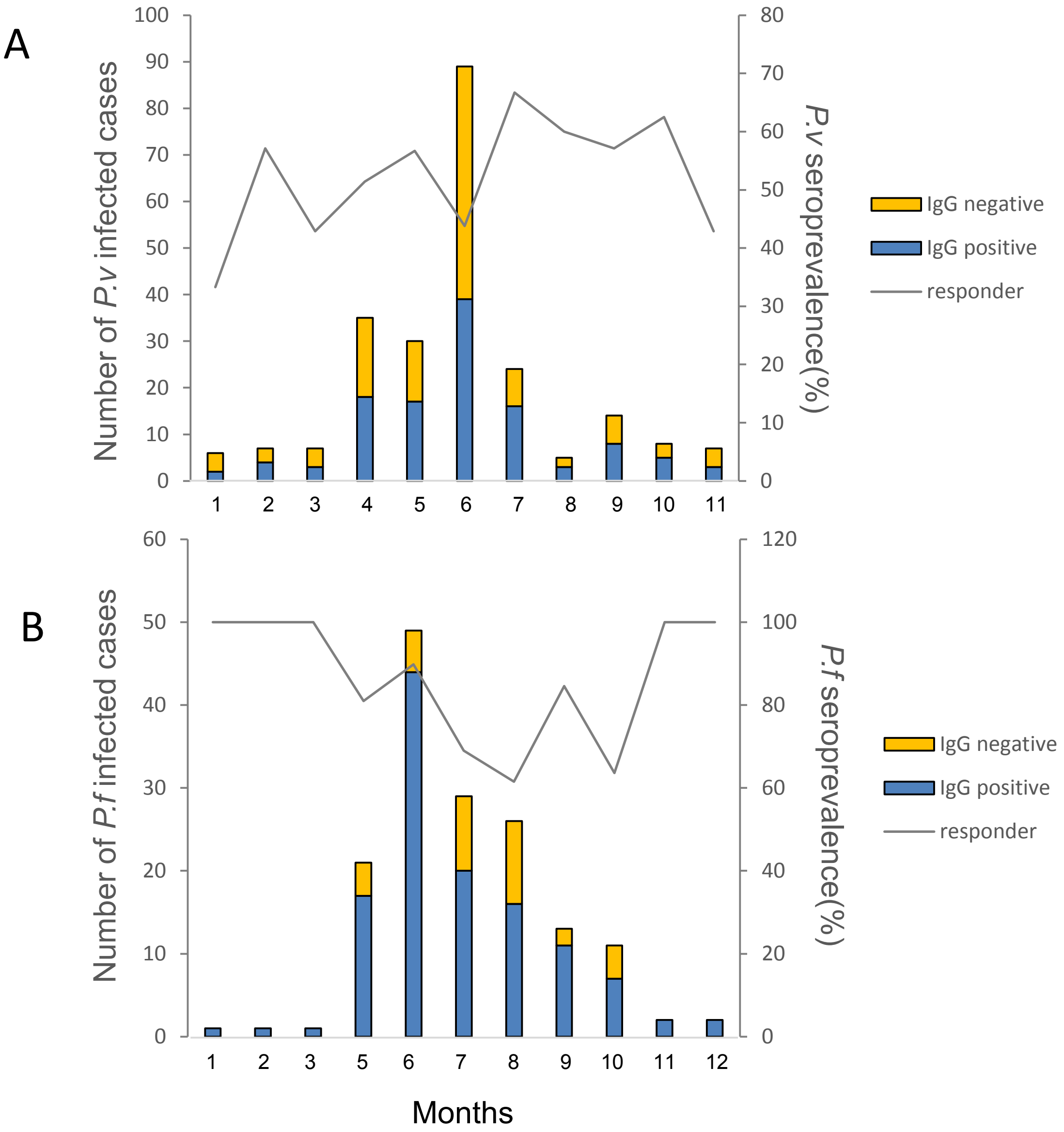

S3 Fig. Distribution and seroprevalence of *P. vivax* (A) and *P. falciparum* (B) patients in different months.
