# Supplementary material for: Naturally Acquired Antibody Responses to Plasmodium vivax and Plasmodium falciparum Merozoite Surface Protein 1 (MSP1) C-Terminal 19 kDa Domains in an Area of Unstable Malaria Transmission in Southeast Asia
Source: PLoS One. 2016 Mar 21;11(3):e0151900. doi: 10.1371/journal.pone.0151900 (PMC4801383; doi:10.1371/journal.pone.0151900)
Supplement: S5 Fig — Data are presented in box plots with the median shown as a line within the box and interquartile value at the edge of box. The range of the column was 1.5 times of interquartile range. Any outlier values exceeding 1.5 and 3 times of the interquartile range are shown as circles and triangles, respectively. Data were analyzed by Mann-Whitney’s U test. (PDF) [file pone.0151900.s005.pdf]

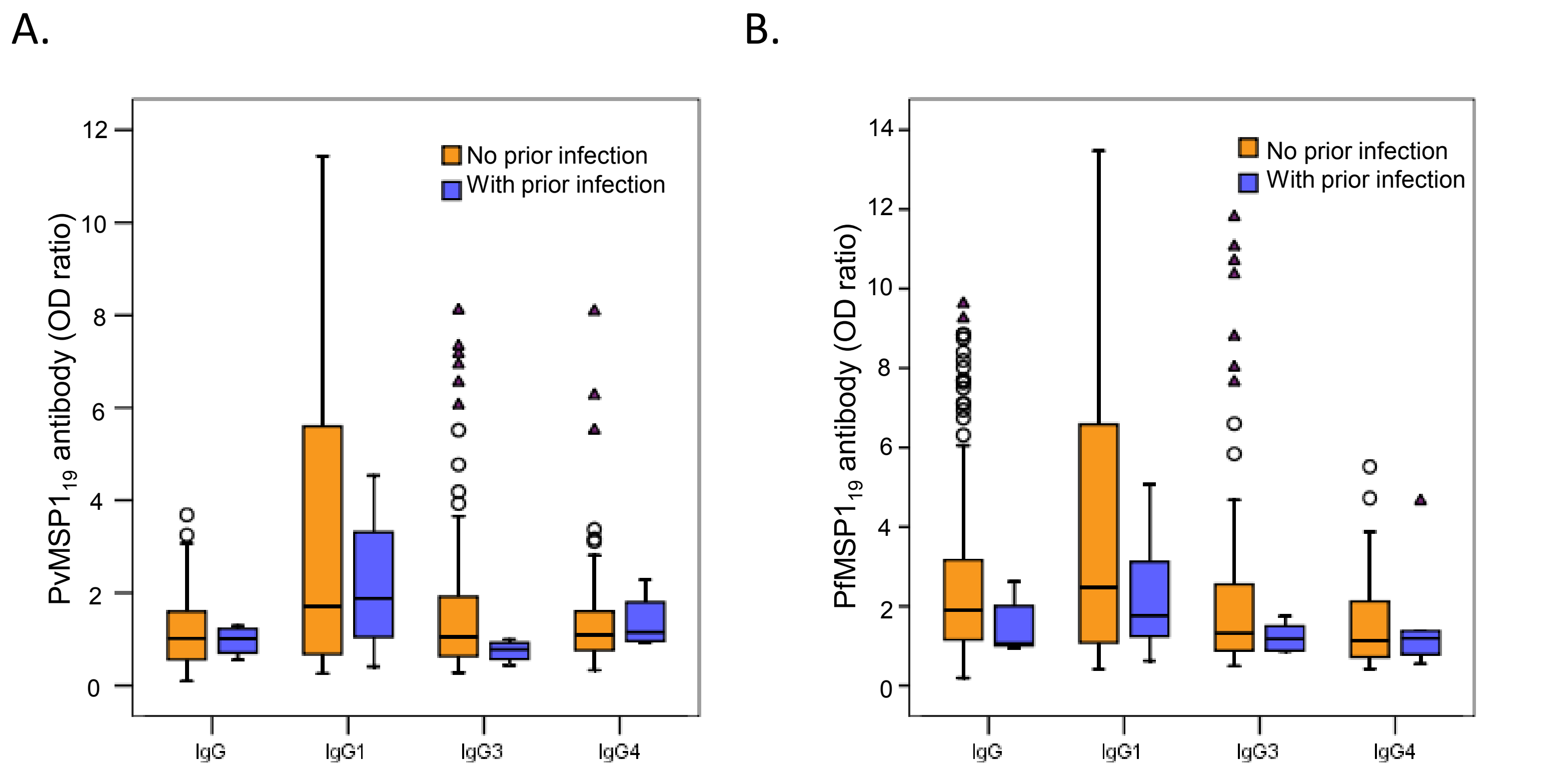

S5 Fig. Antibody responses in acute *P. vivax* (A) and *P. falciparum* (B) infected patients with or without previous *Plasmodium* infection history.
