# Supplementary material for: Naturally Acquired Antibody Responses to Plasmodium vivax and Plasmodium falciparum Merozoite Surface Protein 1 (MSP1) C-Terminal 19 kDa Domains in an Area of Unstable Malaria Transmission in Southeast Asia
Source: PLoS One. 2016 Mar 21;11(3):e0151900. doi: 10.1371/journal.pone.0151900 (PMC4801383; doi:10.1371/journal.pone.0151900)
Supplement: S6 Fig — (A) Alignment of the 5 haplotypes (H1 –H5) with the reference 3D7 sequence. Residue substitutions are shadowed in red. (B) Frequencies of the five haplotypes. (PDF) [file pone.0151900.s006.pdf]

A.

```
3D7           KQCPENSGCFRHLDEREECKCLLNYKQEGDKCVENPNPTCNENNGGCDADATCTEEDSGSSRKKITCECTKPDSYPLFDGIFCSSSNFLGISF
H1  (n=29)    KQCPENSGCFRHLDEREECKCLLNYKQEGDKCVENPNPTCNENNGGCDADAKCTEEDSGSNGKKITCECTKPDSYPLFDGIFCSSSNFLGISF
H2  (n=11)    KQCPENSGCFRHLDEREECKCLLNYKQEGDKCVENPNPTCNENNGGCDADATCTEEDSGSSRKKITCECTKPDSYPLFDGIFCSSSNFLGISF
H3  (n=3)     KQCPQNSGCFRHLDEREECKCLLNYKQEGDKCVENPNPTCNENNGGCDADAKCTEEDSGSNGKKITCECTKPDSYPLFDGIFCSSSNFLGISF
H4  (n=1)     KQCPENSGCFRHLDEREECKCLLNYKQEGDKCVENPNPTCNENNGGCDADATCTEEDSGSNGKKITCECTKPDSYPLFDGIFCSSSNFLGISF
H5  (n=1)     KQCPQNSGCFRHLDEREECKCLLNYKQEGDKCVENPNPTCNENNGGCDADAKCTEEDSGSNGKKITCECTKPDSYPFFDGIFCSSSNFLGISF
              ****:**********************************************.********. **************:****************
```

B.

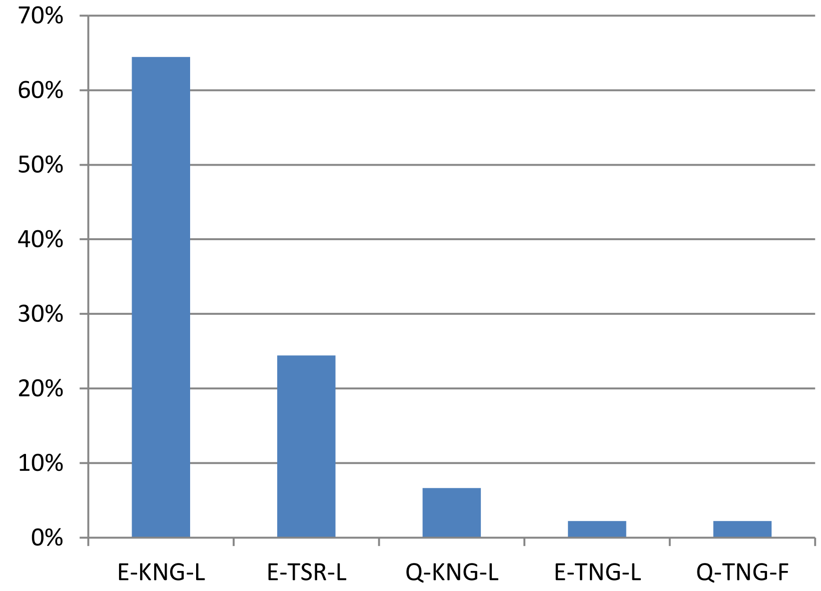


**S6 Fig. PfMSP1$_{19}$ amino acid sequences from 45 available *P. falciparum* samples. (A)** Alignment of the 5 haplotypes (H1 – H5) with the reference 3D7 sequence. Residue substitutions are shadowed in red. **(B)** Frequencies of the 5 haplotypes.
